# Reduction of endoglin receptor impairs mononuclear cell-migration

**DOI:** 10.37349/emed.2020.00010

**Published:** 2020-06-29

**Authors:** Zhenying Han, Sonali Shaligram, Marie E. Faughnan, Dewi Clark, Zhengda Sun, Hua Su

**Affiliations:** 1 Department of Anesthesia and Perioperative Care, University of California, San Francisco, CA 94143, USA; 2 Center for Cerebrovascular Research, University of California, San Francisco, CA 94143, USA; 3 Toronto HHT Centre, Division of Respirology, Department of Medicine, St. Michael’s Hospital, University of Toronto, Ontario M5B 1W8, Canada; 4 Li Ka Shing Knowledge Institute, St. Michael’s Hospital, University of Toronto, Ontario M5B 1W8, Canada; 5 Department of Radiology, University of California, San Francisco, CA 94143, USA

**Keywords:** Endoglin, ALK1, hereditary hemorrhagic telangiectasia, mononuclear cells, migration, oxidative stress

## Abstract

**Aim:**

To test if the impairment of mononuclear cell (MNC) migration in patients with hereditary hemorrhagic telangiectasia (HHT) is due to the reduction of the endoglin (ENG) receptor on the cell surface and oxidative stress.

**Methods:**

MNCs of HHT patients and normal controls were subjected to migration assay. Fractions of MNCs were pre-incubated with antibodies specific to HHT causative genes ENG [hereditary hemorrhagic telangiectasia type 1 (HHT1)] or activin receptor-like kinase 1 [ALK1, hereditary hemorrhagic telangiectasia type 2 (HHT2)], AMD3100 or Diprotin-A to block ENG, ALK1 C-X-C chemokine receptor 4 (CXCR4) or CD26 (increased in HHT1 MNCs) before migration assay. The MNCs were allowed to migrate toward stromal cell-derived factor-1α (SDF-1α) for 18 h. The expression of *CXCR4*, *CD26*, superoxide dismutase 1 (SOD1) and glutathione peroxidase 1 (*GPX1*) in MNCs and nitric oxide levels in the plasma were analyzed.

**Results:**

Compared to the controls, fewer HHT1 MNCs and similar number of HHT2 MNCs migrated toward SDF-1α. Diprotin-A pre-treatment improved HHT1 MNC-migration, but had no effect on normal and HHT2 MNCs. Pre-incubation with an anti-ENG antibody reduced the migration of normal MNCs. Diprotin-A did not improve the migration of ENG antibody pre-treated MNCs. Anti-ALK1 antibody had no effect on MNC-migration. AMD3100 treatment reduced normal and HHT MNC-migration. *ENG* mRNA level was reduced in HHT1 and HHT2 MNCs. *ALK1* mRNA was reduced in HHT2 MNCs only. *CD26* expression was higher in HHT1 MNCs. Pre-treatment of MNCs with anti-ENG or anti-ALK1 antibody had no effect on *CD26* and *CXCR4* expression. The expression of antioxidant enzymes, *SOD1*, was reduced in HHT1 MNCs, which was accompanied with an increase of ROS in HHT MNCs and nitric oxide in HHT1 plasma.

**Conclusions:**

Reduction of ENG receptor on MNC surface reduced monocyte migration toward SDF-1α independent of CD26 expression. Increased oxidative stress could alter HHT MNC migration behavior.

## Introduction

Hereditary hemorrhagic telangiectasia (HHT) or Rendu-Osler-Weber disease is an autosomal dominant vascular disease that affects approximately 1 in 5, 000 people [1–3]. HHT is characterized by the telangiectases on the skin and mucosa, as well as organ arteriovenous malformation (AVMs) and can be complicated by hemorrhage, stroke and heart failure [4]. HHT is classified into hereditary hemorrhagic telangiectasia type 1 (HHT1), hereditary hemorrhagic telangiectasia type 2 (HHT2), and JP (juvenile polyposis)-HHT, depending on the causative gene mutations. Endoglin (*ENG*, HHT1) and activin receptor-like kinase 1 (*ALK1*, HHT2) are major causative genes for HHT, with at least 90% of HHT patients having a mutation in one of these genes [5]. The AVM phenotype in HHT patients and mouse models, e.g., in the skin [6] and in the brain [7–9] is associated with an increased burden of inflammatory cells. AVMs in HHT likely develop as an abnormal response to injury; the role of inflammation in this proposed mechanism remains undefined.

There is evidence that *ENG*-deficiency impairs monocyte homing to the injury site [10]. Recruitment of human monocytes to the infarcted murine heart and subsequent vessel formation are severely impaired when HHT1 monocytes are used [11]. Decreased homing is linked with impaired ability of the monocytes to respond to stromal cell derived factor-1α (SDF-1α). The mechanism has been attributed to increased CD26, also known as dipeptidyl peptidase-4, expression in HHT1 monocytes, a moiety that cleaves the amino-terminal dipeptide from SDF-1α interfering with C-X-C chemokine receptor 4 (CXCR4) binding [10]. Inhibition of CD26 restores the homing ability of HHT1 monocytes. Interestingly, *Eng*-deficiency in endothelial cells reduces leukocyte adhesion and transmigration [12] and impairs the endothelial cell-autonomous capacity to up-regulate SDF-1α expression in response to hindlimb ischemic injury [13]. Although underpowered to detect a significant difference, we also found that there is a 10% reduction in monocyte homing to the angiogenic focus in mice with *Eng*^+/−^ bone marrow (BM) compared to mice with wild-type BM, two weeks after injection of an adeno-associated viral vector expression vascular endothelial growth factor [14]. Taken together, *Eng* deficiency appears to impair monocyte adhesion and migration. Whether loss of *ALK1* affects these processes is not known.

It remains unclear why AVM lesions demonstrate more macrophages if *ENG*-deficiency impairs monocyte migration. We found *Eng*^+/−^ mice had fewer cluster of cluster of differentiation 68^+^ (CD68^+^) microglia/macrophages 3 days after ischemic stroke and more CD68^+^ microglia/macrophages 60 days after stroke in the peri-infarct areas [15]. We have also showed that after angiogenic stimulation, the *Eng* deficient mice had fewer CD68^+^ microglia/macrophages at 2 weeks, similar numbers at 4 weeks, and more at 8 weeks in the brain angiogenic region compared with wild-type mice. These data suggest that delayed macrophage clearance or prolonged microphage infiltration could be a mechanism. The chemotactic activity of SDF-1α not only involves CD26, but also the upregulated expression and activity of integrin family members, which involved in cell adhesion [16–18]. It is not clear if integrin family play roles in the defect of HHT monocyte migration. However, above data indicate that blocking delayed macrophage homing to an AVM lesion could be a therapeutic strategy to reduce AVM severity.

In addition, we have also found that in an *in vitro* angiogenic niches mimicked by the endothelial cells and vascular smooth muscle co-culture system, HHT CD34^+^ mononuclear cells (MNCs) are more likely to be differentiated into macrophages than normal CD34^+^ MNCs, suggesting a pro-inflammatory phenotype of HHT MNCs [9].

In this study, we tested if blocking ENG or ALK1 receptor on the surface of MNC reduces MNC chemotactic migration and if HHT MNCs have a defect in removing oxidative species. Most MNCs used in this study were isolated using Ficoll (StemCell Technology, Vancouver, Canada) density gradient centrifugation, which contain lymphocytes and monocytes. Previous studies showed that only 10–20% MNCs were monocytes, upon activation, both monocytes and lymphocytes expressed ENG protein at the cell surface [19–21].

## Materials and methods

### Isolation of MNCs

Blood collected from HHT patients (Table 1), 15 with known *ENG* mutation (HHT1), 11 with known *ALK1* mutation (HHT2) and 10 with unknown mutations [used for analyzing reactive oxygen species (ROS) in HHT MNCs] were used in this study. Healthy age- and gender-matched volunteers (*N* = 29) were used as controls. Patients and controls were recruited at the Toronto HHT Centre, an HHT Centre of Excellence at St. Michael’s Hospital and the University of Toronto. All patients provided written, informed consent and the study protocol was approved by the Research Ethics Board of St. Michael’s Hospital. We have also purchased concentrated normal monocytes (Trima residuals) from Blood Center of the Pacific (San Francisco, CA).

MNCs that contain lymphocytes and 10–20% monocyte were isolated from 50 mL of blood by Ficoll density gradient centrifugation. Blood were diluted two times with phosphate buffered saline (PBS) plus 2% fetal bovine serum, laid on top of Ficoll and centrifuged at room temperature (15–25°C) for 30 min at 400 × g with brake off. The layer of MNCs was transferred to a sterile 15 mL centrifuge tube. Three volumes of PBS was added to resuspend the cells. The cell suspension was then centrifuged at 400 g for 10 min at 20°C. After removing the supernatant, MNCs were resuspended in 6 mL to 8 mL PBS and centrifuged at 400 g for 10 min at 20°C. After removing the supernatant, MNCs were suspended in cell culture medium (RPMI 1640 Glutamax medium, supplemented with 10% FBS) for migration assay, ROS assay or frozen for quantitative reverse transcription polymerase chain reaction (RT-PCR) analysis. MNCs collected from different HHT patients were used for migration assay and ROS assay (Table 1).

### Migration assay

The membrane of the insert and the bottom of the transwells that were used for migration assay were pre-coated with human fibronectin (20 g/mL, ThermoFisher Scientific, Waltham, MA). Migration medium (RPMI 1640 from ThermoFisher Scientific, supplemented with 0.5% BSA from Sigma Aldrich, St Louse, MO) was added to the inserts of transwells, and incubated at 37°C for 10 min to equilibrate the temperature. The inserts were then transferred to the lower chambers that contained SDF-1α (200 ng/mL, Sigma Aldrich). MNCs were seeded to the insert at a density of 1 × 10^5^/cm^2^ in 100 μL migration medium and allowed to migrate for 18 h at 37°C. Transwell inserts were removed. The excess medium on the bottom and side of the inserts was scraped into the bottom chambers. Cells in the lower chambers were resuspended through pipetting 20 times and then transferred to tubes. The lower chambers were washed with 300 μL PBS once. The PBS was added to tubes with cells collected from the bottom chamber. The tubes containing migrated cells were span at 1, 000 rpm for 5 min. After removing the supernatant, the cells were resuspended with 200 uL PBS and counted using a hemocytometer.

To test the effect of blocking cell surface ENG or ALK1 receptor, prior to the migration assay, MNCs were incubated with an anti-ENG antibody (10 μg/mL, Abcam, Cambridge MA) or an anti-ALK1 antibody (10 μg/mL, R&D Systems, Minneapolis, MN) for 1 h in migration medium. After incubation, the antibody-containing media were removed and the cells were replenished with fresh migration media.

As controls, MNCs were incubated with AMD3100 (5 μg/m, Sigma Aldrich) for 30 min to block CXCR4 receptor or Diprotin-A (5mM, Sigma Aldrich) for 15 min to block CD26 prior migration assay.

The phenotype of migrated cells was analyzed by fluorescent-activated cell sorting (FACS) using macrophage markers: CD11b and CD14, endothelial marker: CD31, and pan lymphocyte marker: CD3. Antibodies used in the FACS were anti CD14-PE-Cy7 (1:100), anti-CD11b-V450 (1:100), anti CD31-V450 (1:100), and anti CD3-FITC (1:100). All antibodies were purchased from BD Biosciences (San Jose, CA). BD CompBead Anti-Mouse Ig Kappa (BD Biosciences) incubated with each antibody was used to optimize fluorescence compensation settings for multicolor flow cytometric analysis. The samples were analyzed on a FACS LSR II (BD Biosciences), and percentage of each antiboded stained cells was calculated using DIVA V 6.1.3 software.

### Quantitative RT-PCR analysis

The mRNA levels of *CD26*, *CXCR4*, *ENG*, *ALK1*, and two antioxidant enzymes, superoxide dismutase 1 (SOD1) and GPX1 in MNCs were quantified by quantitative real-time-PCR. Total RNA was extracted from MNCs using RNAzol@RT (Molecular Research Center, Cincinnati, OH) and reverse-transcribed into cDNA using SuperScript* III First-Strand Synthesis System (Invitrogen, Carlsbad, CA). Real-time PCR was performed using TaqMan Fast Advanced Master Mix (Applied Biosystems, Foster City, CA). Gene-specific primers and probes purchased from Applied Biosystems were used: *CD26* (Hs00897386_m1), *CXCR4* (Hs00607978_s1), ALK1 (ACVRL1, Hs00953798_m1), *ENG* (Hs00923996_m1), SOD1 (Hs00533490_m1), GPX1 (Hs01028922_g1), *HPRT* (Hs02800695_m1), and GAPDH (Hs02758991_g1). All samples were run in duplicates or triplicate, and relative gene expression was calculated using the comparative threshold cycle (CT) and normalized to *GAPDH* or *HTRT* (ΔCT).

### Measurement of mitochondrial ROS level in MNCs and nitric oxide level in plasma

The blood cells and plasma were separated by centrifugation (3, 000 g for 10 min). CD34^+^ monocytes were isolated using CD34-antibody-coated Dynabeads (Life Technologies, South San Francisco, CA) according to manufacturer’s instructions. Total ROS in CD34^+^ MNCs of HHT patients were measured using the Total ROS Detection Kit (Thermo Scientific) following the manufacturer’s protocols. ROS positive cells and fluorescent intensity were quantified by microscopic analysis. NOx in the plasma of HHT patients using Nitric Oxide Assay kit (Thermo Scientific) following the manufacturer’s protocols.

### Statistical analysis

Data are represented as mean ± SD. Prism 6 (GraphPad Software Inc., La Jolla, CA) was used for all the statistical analyses in this study. Sample sizes are shown in figure legends. Two-way analysis of variance (ANOVA) followed by Tukey’s post hoc test was used to compare the differences of MNC migration among groups. One-way ANOVA followed by Tukey’s post hoc test was used to analyze the differences of gene expression. Student *t* test was used to compare the differences when there are two testing groups involved. A *P*-value equals or smaller than 0.05 was considered significant.

## Results

### The number of HHT1 MNCs migration towards SDF-1α is reduced

**Figure 1.**
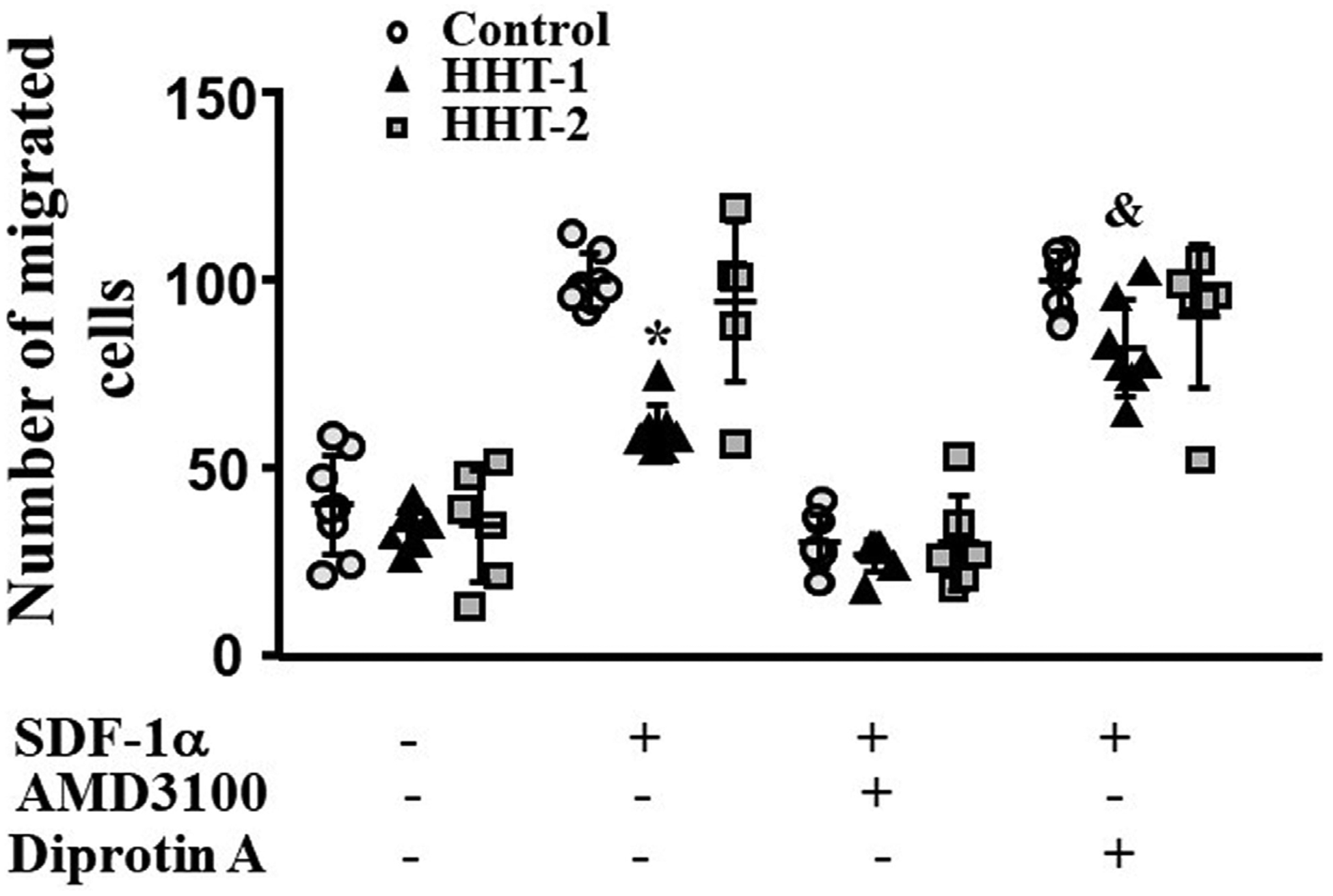
Reduction of HHT1 MNC migration toward SDF-1α. *N* = 8 for control MNCs; *N* = 7 for HHT1 MNCs; and *N* = 6 for HHT2 MNCs. *: *P* < 0.001 compared to control; &: *P* = 0.005 compared to untreated HHT-1

MNCs were isolated from the blood of HHT1 and HHT2 patients, and age and sex matched controls. MNCs migration towards SDF-1α was analyzed in a transwell system. Two-way ANOVA analysis showed that the difference among groups was significant (*P* < 0.001). Compared to controls (100.0 ± 7.0 cells), there were fewer HHT1 MNCs (60.3 ± 6.8 cells) migrated to the lower chambers (where the media contained SDF-1α, *P* < 0.001, Tukey’s multiple comparisons). Similar numbers of HHT2 (94.5 ± 21.2 cells) and normal MNCs migrated into the lower chambers (*P* = 0.23). Blocking CXCR4 receptor by pre-treating the cells with AMD3100 was equally effective in blocking HHT1, HHT2 and normal MNCs migration toward SDF-1α (*P* < 0.001 vs. untreated cells). Block of CD26 through Diprotin-A pre-treatment increased the number of HHT1 MNCs in lower chambers (82.0 ± 13.0, *P* < 0.001). Diprotin-A had no effect on normal and HHT2 MNCs (90.5 ± 19.1 cells, *P* = 0.94, Figure 1). Therefore, MNC-migration toward SDF-1α is impaired in HHT1, but this can be rescued by blocking CD26 receptor.

### Blocking ENG receptor on MNC-surface reduce their migration toward SDF-1α

**Figure 2.**
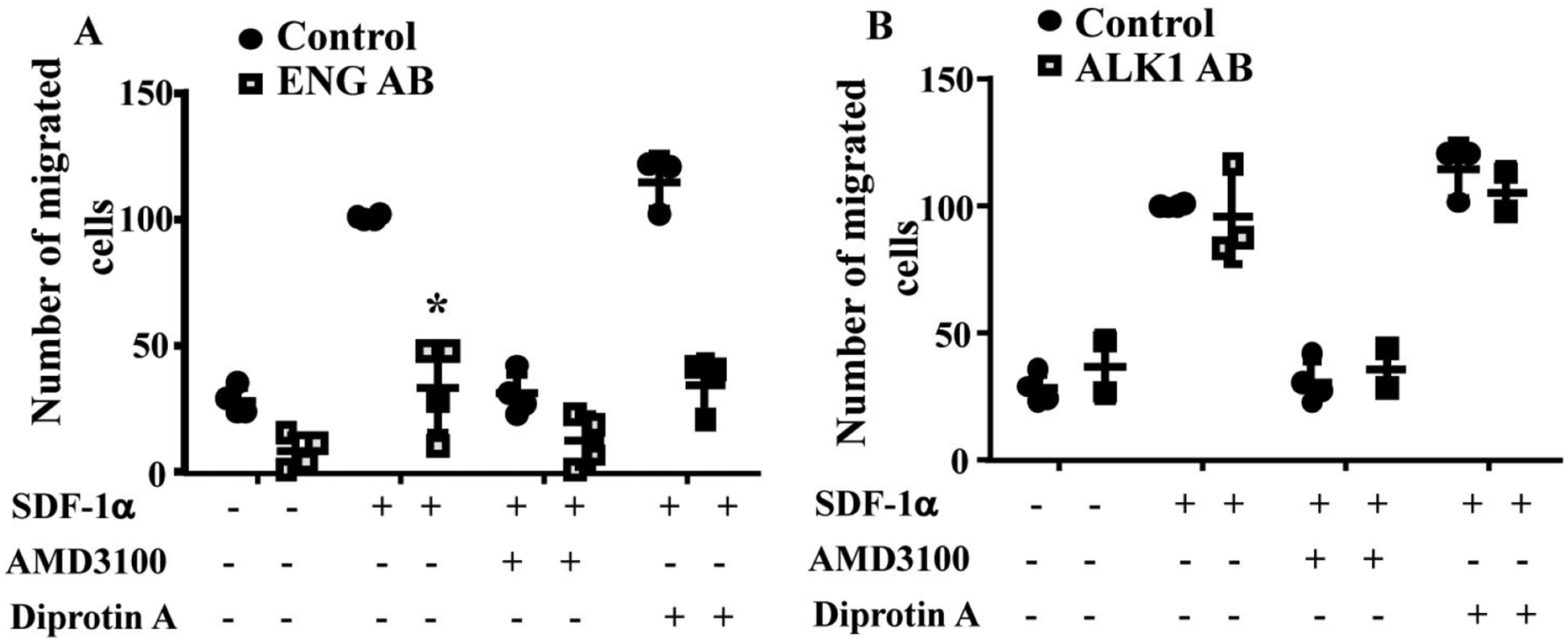
ENG antibody (ENG AB) pre-treatment reduced MNC-migration toward SDF-1α. A. Quantification of the migration of ENG antibody pre-treated normal MNCs. Controls are MNCs pretreated with corresponding control IgG. *: *P* < 0.001 *versus* control IgG treated cells. B. Quantification of the migration of ALK1 antibody pre-treated normal MNCs. Controls are MNCs pretreated with corresponding control IgG. *N* = 4 for control IgG treated groups; *N* = 4 for ENG antibody treated groups; and *N* = 3 for ALK1 antibody (ALK1 AB) treated groups

To test if blocking ENG or ALK1 receptor on MNC-surface could influence MNC migration toward SDF-1α, MNCs isolated from the concentrated normal monocytes (Trima residuals) purchased from Blood Center of the Pacific (San Francisco, CA) were incubated with an anti-ENG antibody or an anti-ALK1 antibody before being subjected them to migration assay. Two-way ANOVA analysis showed that the difference among groups are significant (*P* < 0.001). Compared to MNCs pretreated with control IgG (100.5 ± 0.9 cells), there were fewer ENG antibody pretreated MNCs (33.6 ± 18.1cells) migrated to lower chambers (*P* < 0.001, Tukey’s multiple comparisons test, Figure 2A). Reduction of CD26 by Diprotin A pretreatment did not improve the migration ability of ENG antibody treated MNCs (*P* > 0.999). Pre-treatment of MNCs with ALK1 antibody did not affect MNC migration toward SDF-1α (*P* = 0.998, Figure 2B). These data indicate that the availability of ENG receptors on MNC-surface is crucial for MNC migration toward SDF-1α.

**Figure 3.**
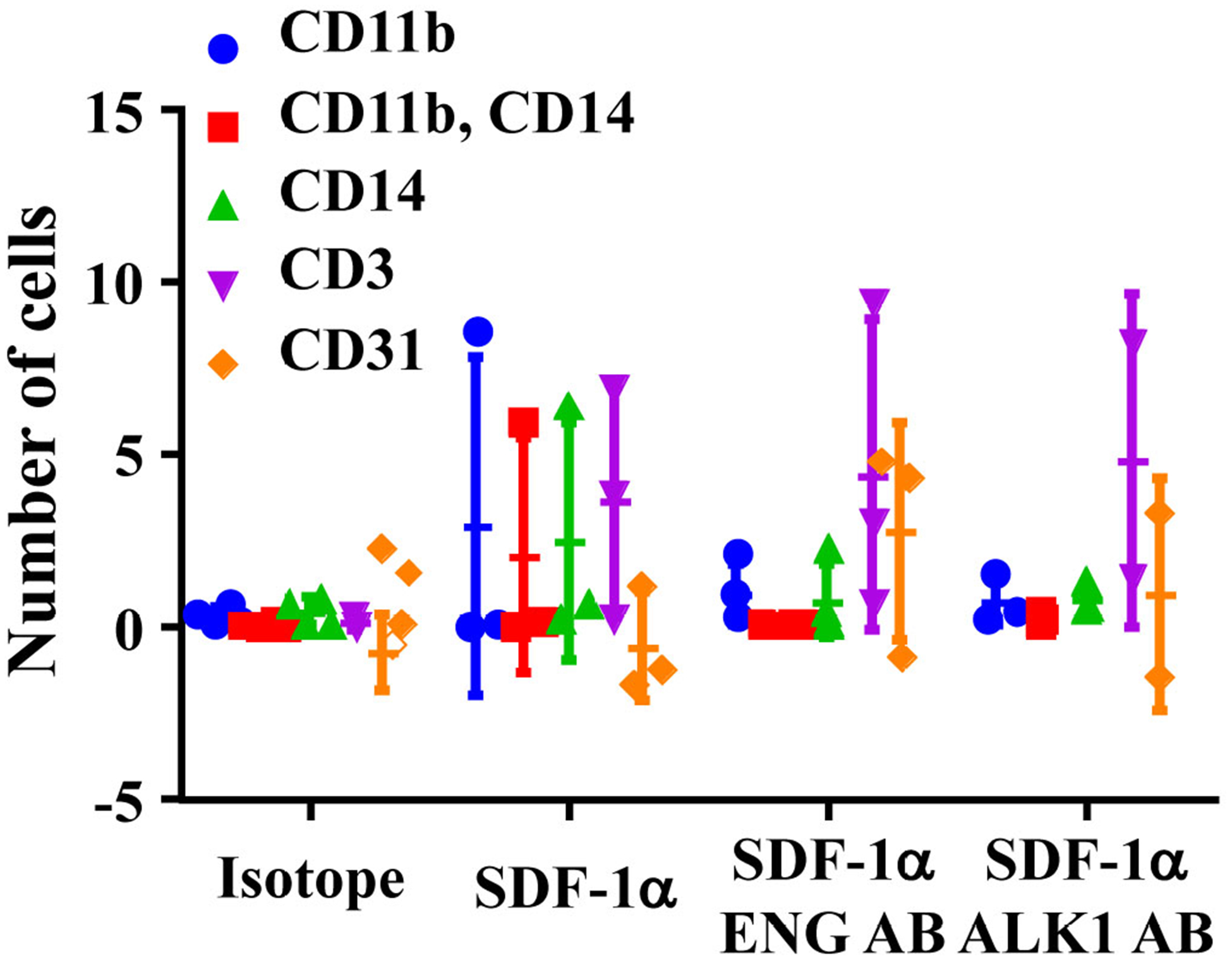
ENG antibody (ENG AB) or ALK1 antibody (ALK1 AB) pre-treatment reduced monocyte-migration toward SDF-1α. Isotope: MNCs pretreated with corresponding IgGs for ENG AB and ALK1 AB. SDF-1α: SDF-1α was added to bottom chamber in migration assay; SDF-1α ENG AB or SDF-1α ALK1 AB: MNCs pre-treated with ENG or ALK1 antibody and with SDF-1α in the bottom chamber

The phenotypes of migrated cells were analyzed by FACS. Monocytes were detected by antibodies specific to CD11b and CD14. Lymphoycytes were detected by an antibody specific to CD3. Same numbers of monocytes (CD11b^+^, or CD14^+^, or CD11b^+^+CD14^+^) and lymphocytes (CD3^+^) were migrated toward to SDF-1α. Interestingly, ENG antibody or ALK1 antibody treatment reduced monocytes migration, but have no effect on lymphocyte-migration (Figure 3). Due to the large variation and limited sample sizes, we could not detect any difference among groups.

### Pre-treatment of MNCs with anti-ENG or anti-ALK1 antibody had no effect on CD26 and CXCR4 expression

**Figure 4.**
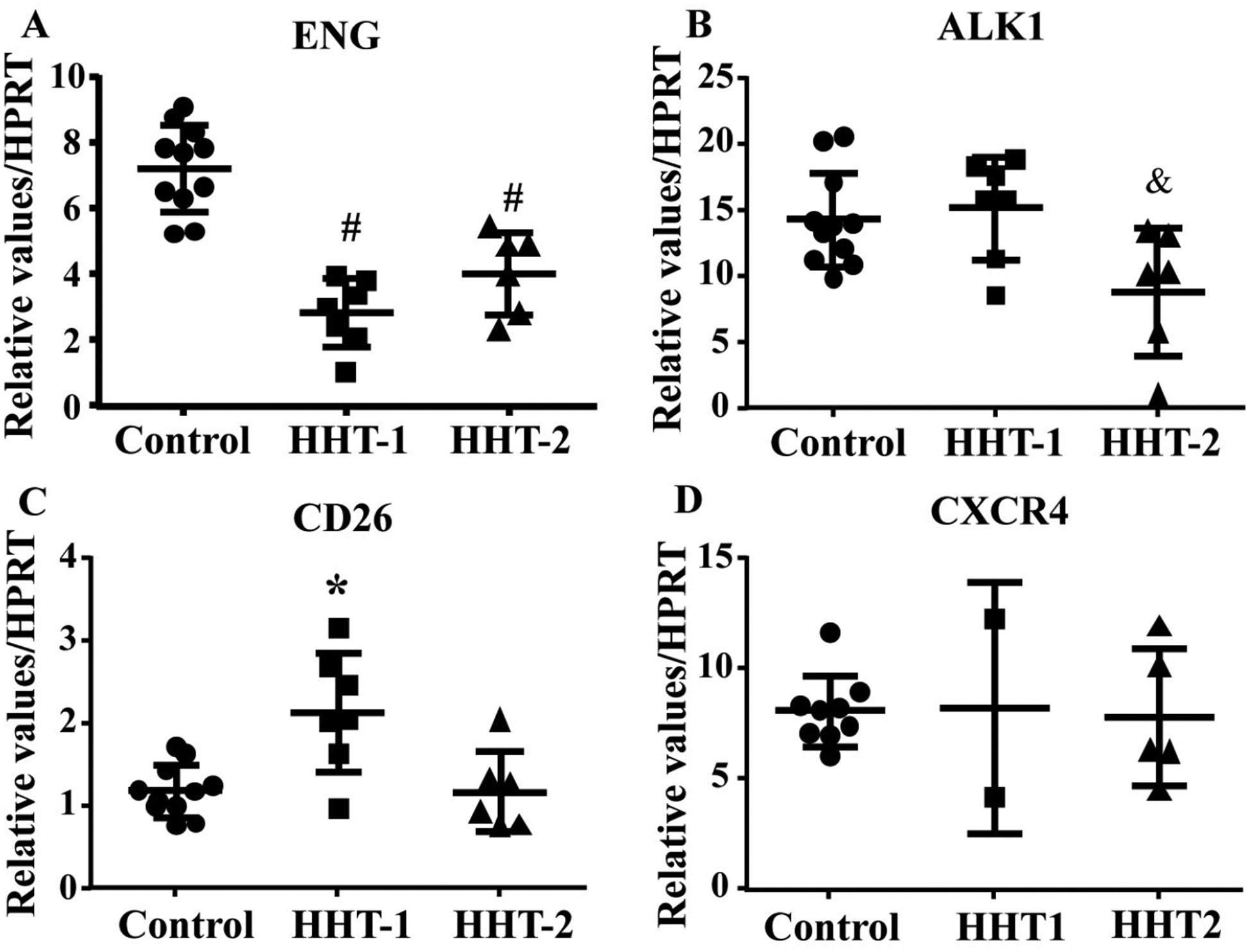
*CD26* expression is increased in HHT1 MNCs. A. Quantification of *ENG* mRNA. #: *P* < 0.001 compared to control; #: *P* < 0.001 compared to control. B. Quantification of ALK1 mRNA. &: *P* = 0.03 compared to control. C. Quantification of *CD26* mRNA. *: *P* = 0.002 compared to control. D. Quantification of *CXCR4* mRNA. For A, B and C, *N* = 11 for control, *N* = 7 for HHT1 and *N* = 6 for HHT2. For D, *N* = 5 for control; *N* = 2 for HHT1; and *N* = 4 for HHT2

To test if antibody treatment influences the expression of *CD26* and *CXCR4*, the mRNA levels of these genes were analyzed by quantitative RT-PCR. As control, the expression of these genes in HHT1 and HHT2 MNCs were analyzed first. Similar to published data [10, 22], *ENG* gene expression was reduced in HHT1 (*P* < 0.001) and HHT2 (*P* < 0.001) MNCs (Figure 4A). *ALK1* gene expression was reduced in HHT2 MNCs (*P* = 0.03, Figure 4B). *CD26* gene expression was significantly increased in HHT1 MNCs only (HHT1 *versus* control: *P* = 0.002; HHT2 *versus* control: *P* = 0.0997, Figure 4C). *CXCR4* expression was not significantly different among control, HHT1 and HHT2 MNCs (*P* = 0.97, Figure 4D).

**Figure 5.**
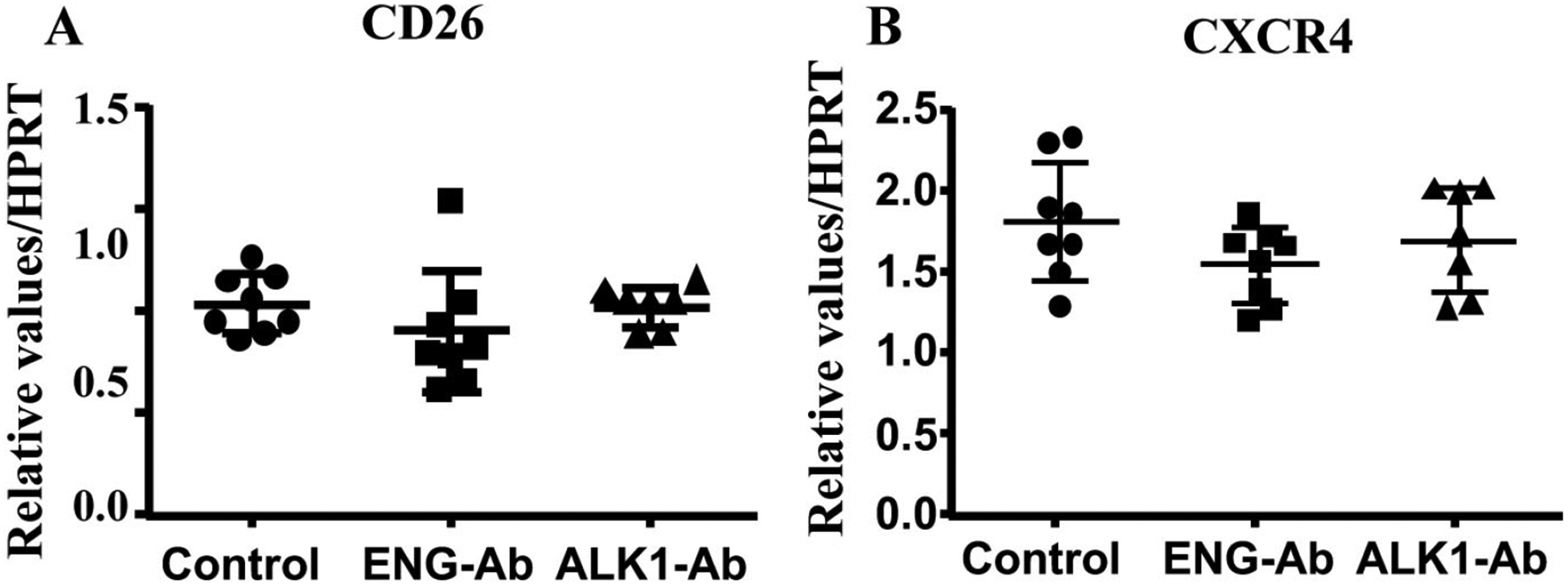
ENG or ALK1 antibody (Ab) treatment did not alter *CD26* and *CXCR4* expression. A. Quantification of *CD26* expression. B. Quantification of *CXCR4* expression. *N* = 8 for control; *N* = 8 for ENG-Ab group; *N* = 7 for ALK1-Ab treated group. Control: corresponding control IgG treated MNCs; ENG-Ab: ENG antibody treated MNCs; ALK1-Ab: ALK1 antibody treated MNCs

We next analyzed *CXCR4* and *CD26* expression in ENG or ALK1 antibody treated MNCs. We found ENG or ALK1 antibody treatment did not alter the expression of CD26 (*P* = 0.34) and *CXCR4* (*P* = 0.24, Figure 5).

### Antioxidant enzyme is reduced and ROS is increased HHT MNCs, which are associated with an increase of NOx in the plasma of HHT patients

**Figure 6.**
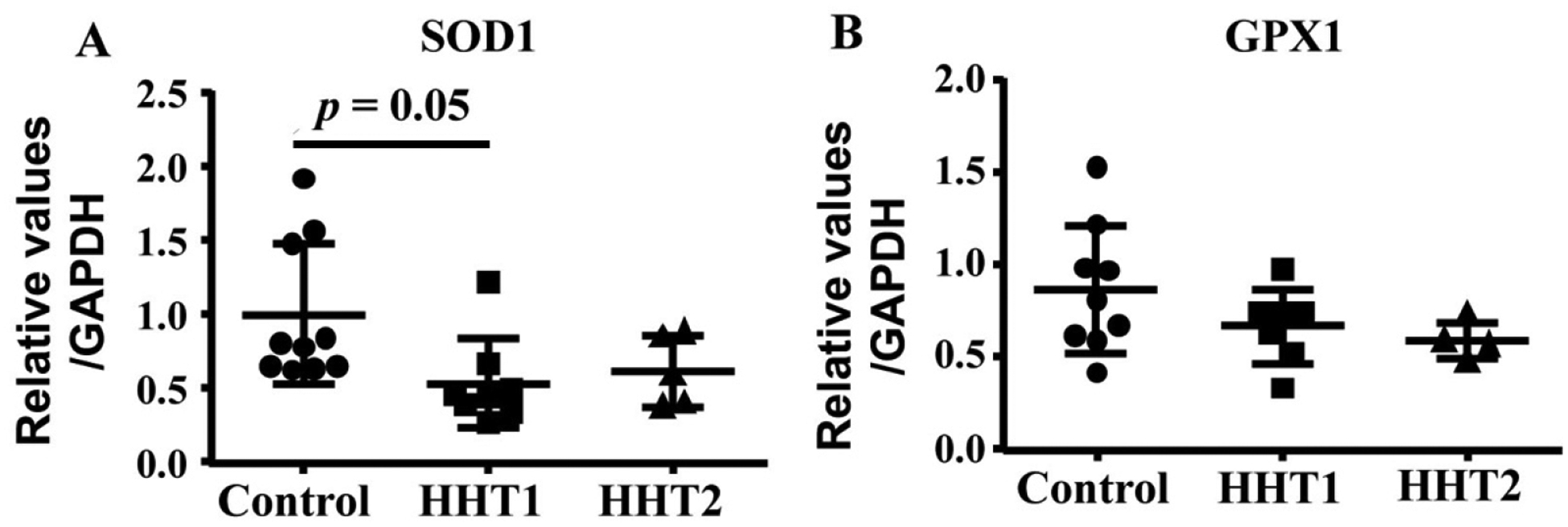
HHT1 MNCs expressed lower levels of *SOD1* (superoxide dismutase 1). A. Quantification of *SOD1* mRNA. *N* = 10 for control group; *N* = 8 for HHT1 group; and *N* = 5 for HHT2. B. Quantification of *GPX1* (glutathione peroxidase 1) mRNA. *N* = 10 for control group; *N* = 7 for HHT1 group; and *N* = 4 for HHT2 group

We showed previously that HHT MNCs are more likely to differentiate into pro-inflammatory macrophages in angiogenic environment than normal MNCs in an *in vitro* angiogenic niche [9]. Macrophage mitochondrial oxidative stress has been shown to promote nuclear factor-κB-mediated inflammation in macrophage [23]. To test if HHT MNCs pro-inflammatory phenotype have any association with oxidative stress, we analyzed the mRNA levels of antioxidant enzymes, SOD1 and GPX1. ANOVA analysis showed that the *SOD1* level was different among HHT1, HHT2 and control MNCs (*P* = 0.04). Multiple comparison showed that HHT1 MNCs expressed lower levels of *SOD1* than control MNCs (*P* = 0.05). The difference between HHT2 MNCs and control MNCs (*P* = 0.19), HHT1 and HHT2 (*P* = 0.92) did not reach statistic significant. Of note, the lack of difference between HHT2 and control MNCs could be due to the small sample size (*N* = 5) of HHT2 MNCs. ANOVA analysis did not show significant difference of *GPX* level among HHT1, HHT2 and control MNCs (*P* = 0.19, Figure 6).

**Figure 7.**
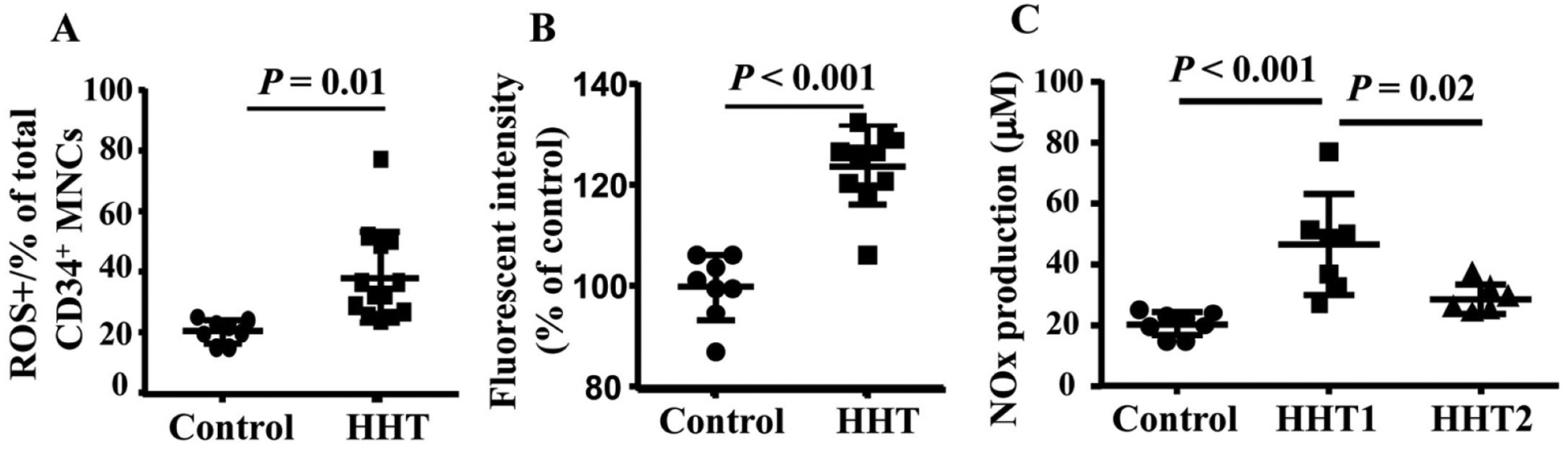
Increased ROS in HHT MNCs and HHT plasma. A. Quantification of ROS^+^ CD34^+^ monocytes. B. Quantification of fluorescent intensity in CD34^+^ monocytes. For A & B analyses, *N* = 8 for control group; *N* = 10 for HHT group. C. Quantification of NOx in plasma. *N* = 8 for control group; *N* = 7 for HHT1 group; and *N* = 6 for HHT2 group

Interestingly, we found that the ROS was increased in HHT MNCs (*P* = 0.01 for ROS^+^ cells and *P* < 0.001 for fluorescent intensity, Figure 6A and B). Since these patients were not genotyped, it is not clear if HHT1 and HHT2 MNCs have different levels of ROS. The NOx (NO2^−^ and NO3^−^) level is increased in the plasma of HHT1 patients (Figure 6C) compared to the plasma collected from controls (*P* < 0.001) and HHT2 (*P* = 0.02). NOx level in HHT2 and control plasma had no difference (*P* = 0.3, Figure 7). These data suggest that reduced levels of antioxidant enzyme in HHT MNCs reduce their ability to remove oxidative species in MNCs and plasma, which may contribute to a pro-inflammatory phenotype.

## Discussion

It has been hypothesized that AVM formation involves an impaired response to injury that may include the dysfunction of peripheral blood mononuclear cells [22, 24]. The human AVM phenotype in HHT in the skin [6] and in the brain [7, 25, 26] is associated with an increased burden of inflammatory cells. Brain AVM lesions in HHT mouse models also have many macrophages [8, 9, 25]. However, there is evidence that *ENG*-deficiency reduces monocyte homing to the injury site [10, 11], which was linked with impaired ability of the monocytes to respond to SDF-1α.

Before MNCs are able to contribute to repair, they need to home to and retain in ischemic and damaged tissue. Directed migration (homing) of MNCs following tissue damage is regulated by the SDF-1α. MNCs that express the CXCR4 migrate toward the tightly regulated gradient of SDF-1α. This directed migration of monocytes can be inhibited by CD26, a moiety that cleaves the amino-terminal dipeptide from SDF-1α interfering with CXCR4 binding [10]. MNCs of HHT1 patients express elevated levels of CD26 and show impaired homing toward damaged tissue [24]. Impaired homing capacity of the MNCs might therefore contribute to the impaired angiogenesis and tissue repair observed in HHT1 patients due to impaired resolution of inflammation [27]. However, if *ENG*-deficiency reduces monocyte migration, it is unclear why AVM lesions contain more macrophages. We have previously demonstrated a pro-inflammatory phenotype of HHT1 MNCs with delayed and prolonged MNC homing and delayed clearance [9, 28], which could be the underlying mechanisms for macrophages accumulated in AVM lesions.

Although ENG expression is reduced in HHT2 MNCs, their migration toward SDF-1α was not significantly reduced. The reduced migration of HHT1 MNCs has been attributed to their increased expression of CD26. However, CD26 level is not increased in HHT2 MNCs. The low CD26 levels on HHT2 MNCs did not correlate with their migratory behavior capacity [10]. Another explanation could be the small sample size and large variation of HHT2 MNCs prevent us from detecting the defect. The migration ability of HHT2 MNCs should be investigated further in future study.

In this paper, we demonstrated that reduction of available ENG receptors on MNC-surface by specific antibody treatment reduced normal MNC-migration toward SDF-1α. The antibody treatment did not change *CD26* and *CXCR4* mRNA expressions in MNCs, which indicate that the antibody effect is independent of CD26 expression. The low CD26 levels on normal MNCs did not correlate with their migratory behavior capacity [10]. Therefore, inhibition of CD26 could not restore the migration of ENG Ab treated MNCs. One limitation here is that we did not analyze the CD26 and CXCR4 protein on cells surface. Pre-treatment of MNCs with anti-ALK1 antibody did not reduce MNC-migration toward SDF-1α, suggesting that ENG may have a direct effect on monocyte migration and the mechanisms underlying the accumulation of monocytes/macrophages in HHT1 and HHT2 AVMs may not be identical and need further study. The chemotactic activity of SDF-1α also upregulate expression and activity of integrin family members, which are involved in cell adhesion [16–18]. The role of integrin family in the defect of HHT monocyte migration should also be investigated in future study.

One clinical study has shown that alveolar exhaled NO is elevated in HHT patients [29]. Uncoupled eNOS activity resulting in impaired myogenic response has been reported to represent an early event in HHT pathogenesis related to oxidative stress [30–33]. Increased levels of free radicals including superoxide, NO, and peroxynitrite have been reported to mediate vascular damage [34–39]. Increased ROS has been demonstrated to be one of the mechanisms for non-resolving inflammation [40], the one mechanism demonstrated to have clear relevance to HHT in both *Eng* [31] and *Alk1* deficiency [32]. However, it was not clear previously that if ROS increases in HHT MNCs and if increased ROS drives monocyte pro-inflammatory phenotype. We demonstrated here that the level of an antioxidant enzyme, SOD1 in HHT MNCs was reduced, which was accompanied with an increase of ROS in HHT MNCs. In addition, the NOx level is also increased in the plasma of HHT patients. Since macrophage mitochondrial oxidative stress have been shown to promote nuclear factor-κB-mediated inflammation in macrophage [23], the reduction of antioxidant enzyme, increase of ROS in HHT MNCs and the NOx in the plasma of HHT patients may contribute to the pro-inflammatory phenotype of HHT MNCs.

The role of inflammation in HHT disease remains poorly understood [24]. The up-regulation of ENG in activated monocytes is reduced in HHT1 and HHT2 patients [41]. Therefore, it can be postulated that *ENG* gene expression might be necessary for proper macrophage performance. This theory is supported by clinical reports of brain abscess, osteomyelitis, septic arthritis, recurrent *Staphylococcus aureus* and other infections in HHT patients [41, 42]. Animal study showed that deletion of *Eng* in macrophages impaired the phagocytic activity of peritoneal macrophages [43], this could help to explain the higher rate if infectious disease seen in HHT patients. As we have discussed in previous sections in this paper that HHT1 monocytes are not able to homing to injury/angiogenic sites in response to SDF-1α immediately after the injury [9–11, 15], which may delay the clearance the damaged tissue. However, they enter continually and stay in the injury/angiogenic site for an extended period leading to an unresolved inflammation and delayed tissue repair [9, 15]. We have also showed previously that HHT MNCs have a pro-inflammatory phenotype. In this study, we demonstrated that HHT MNCs have increased ROS, suggesting that they are undergo oxidative stress. All of these indicate that reduction of ENG in HHT MNCs impairs their response to tissue injury and infection. Large prospective studies are needed to characterize the association of altered HHT MNC-function and risk of infection.

The alternation of signaling pathways in HHT MNCs that are responsible to their altered functions are largely unknown. It has been shown that *ENG* heterozygous endothelial cells exhibit reduced ALK1-Smad1/5 signaling. Unexpectedly, these cells adapted their ALK5 expression with a decrease of 80% and therefore, they also have reduced ALK5-Smad2/3 signaling [44, 45]. We found in this study that ENG expression is reduced not only in HHT1 MNCs but also reduced in HHT2 MNCs. Whether, reduction of ENG on HHT MNCs is the only factor that contribute to the impaired function of HHT MNCs or there is other pathways, such as ALK5 signaling involved need to be study in future.

In summary, we demonstrated here that reduction of the availability of ENG receptor on the surface of MNCs is a mechanism for the reduction of HHT1 MNC-migration to SDF-1α. In addition, HHT MNCs and plasma have reduced anti-oxidant gene expression and increased ROS, which could be responsible for a pro-inflammatory phenotype in HHT patients.

## Figures and Tables

**Table 1.** HHT patients

Patients	Genotype	Mutations	Used in studies
16	*ALK1*	c.586A>G	1. Migration
24	*ALK1*	no information	2. qPCR for *ENG, ALK1, CD26* and *CXCR4*
25	*ALK1*	c.772+2C>G	
32	*ALK1*	c.867_868delinsTT (p.Gln290X)	3. NOx in plasma
33	*ALK1*	c.1, 403T>G (p.Met468Arg)	
36	*ALK1*	c.329C>A (p.Ser110X)	
29	*ENG*	c.690–2A>T	
30	*ENG*	deletion of exon 2	
37	*ENG*	c.1, 205dupA	
38	*ENG* (*ENG* family)	no information	
44	*ENG*	c.115delA	
28	*ENG*	no information	
45	*ENG*	no information	
CT002	*ENG*	c.657–658delCA	qPCR for *SOD1* and *GPX1*
CT003	*ENG*	c.1, 082_1, 085del (p.Thr361Serfs)	
CT004	*ALK1*	c.1, 107_1, 108delAG (p.Arg369fs)	
CT005	*ENG*	c.20dupC (p.Leu8fs)	
CT006	*ENG*	c.38T>G (p.Leu13Arg)	
CT007	*ALK1*	c.1, 231C>T (p.Arg411Trp)	
CT008	*ALK1*	c.1, 120C>T (R374W)	
CT009	*ENG*	Familial mutation	
CT011	*ALK1*	c.430C>T (p.Arg144X)	
CT012	*ENG*	c.588G>A (W196X)	
CT013	*ENG*	c657–658delCA	qPCR for *SOD1*
CT016	*ALK1*	c. 1, 435C>T (p.arg479*)	
CT001	*ENG*	c.655C>G (p.His219Asp)	

qPCR: quantitative polymerase chain reaction; NOx: nitrogen oxide; *GPX1*: glutathione peroxidase 1

## Abbreviations

Ab: antibody
ALK1: activin receptor-like kinase 1
ALK1-Ab: ALK1 antibody treated MNCs
ANOVA: analysis of variance
AVM: arteriovenous malformation
CD68^+^: cluster of differentiation 68^+^
CT: threshold cycle
CXCR4: C-X-C chemokine receptor 4
ENG: endoglin
ENG-Ab: ENG antibody treated MNCs
FACS: fluorescent-activated cell sorting
GPX1: glutathione peroxidase 1
HHT: hereditary hemorrhagic telangiectasia
HHT1: hereditary hemorrhagic telangiectasia type 1
HHT2: hereditary hemorrhagic telangiectasia type 2
MNCs: mononuclear cells
NOx: nitric oxides
PBS: phosphate buffered saline
ROS: reactive oxygen species
RT-PCR: reverse transcription polymerase chain reaction
SDF-1α: stromal cell derived factor-1α
SOD1: superoxide dismutase 1
